# Clinical Characteristics, Preventive Care and Attitude to Telemedicine among Patients with Diabetic Retinopathy: A Cross-Sectional Study

**DOI:** 10.3390/jcm10020249

**Published:** 2021-01-12

**Authors:** Siddarth Agrawal, Bartłomiej Strzelec, Rafał Poręba, Anil Agrawal, Grzegorz Mazur

**Affiliations:** 1 Department and Clinic of Internal Medicine, Occupational Diseases, Hypertension and Clinical Oncology, Wroclaw Medical University, 50-556 Wrocław, Poland; rafal.poreba@umed.wroc.pl (R.P.); grzegorz.mazur@umed.wroc.pl (G.M.); 2 Department of Pathology, Wroclaw Medical University, 50-368 Wrocław, Poland; 3 Second Department and Clinic of General and Oncological Surgery, Wroclaw Medical University, 50-556 Wrocław, Poland; bstrzelec94@interia.pl (B.S.); anil.agrawal@umed.wroc.pl (A.A.)

**Keywords:** telemedicine, diabetic retinopathy, preventive medicine, cross-sectional study

## Abstract

Diabetic retinopathy (DR) is the most frequent and one of the most severe complications of both types of diabetes. Despite the development of versatile diabetes management programs in most developed countries, many patients remain at increased risk for developing this life-limiting and life-threatening condition. This cross-sectional analysis objective was to examine and compare the prevalence of diabetic retinopathy and comorbidities, as well as the clinical characteristics, prevention patterns, and attitude to telemedicine in patients with diabetes. We found that, when compared to the non-DR group, patients with DR significantly more often utilize clinical preventive services and counseling; however, there is still a significant gap in the receipt of preventative care. Moreover, in the DR subgroup, inadequate diabetic control and the presence of various signs and symptoms of diseases were observed. Although less than a fifth of all patients use mobile applications to monitor their health status, the patients indicate their willingness to use telemedical technology, particularly if it is recommended by the physician and provided without additional costs. The evolution of telemedicine offers a possibility of inexpensive, continuous monitoring of the disease that could improve treatment outcomes. Our observations emphasize DR’s perception as a complex disease in which education and continuous monitoring, particularly with telemedicine methods, are critical for further improvement in chronic care.

## 1. Introduction

The global diabetes epidemic correlating with obesity has been observed for several decades. This disturbing phenomenon leads to a significant increase in the prevalence of type 2 diabetes. Additionally, although not fully understood, an increase in type 1 diabetes incidence has been observed [1,2]. In accordance with IDF DIABETES ATLAS Ninth Edition 2019, an estimated 463 million people have diabetes worldwide (8.8% of the adult population); type 2 diabetes is responsible for about 90% of cases. Diabetic retinopathy (DR) is the most frequent and one of the most severe complications of both types of diabetes. DR remains the leading cause of preventable blindness in the middle-age population in developed countries, which leads to severe medical and economic issues [2,3,4,5,6,7]. Despite the development of versatile diabetes management programs in most developed countries, many patients remain at increased risk of developing DR and other neurodegenerative and microvascular complications [2,4,5]. The pathophysiology involves several biochemical mechanisms, which lead to inflammation, local ischemia, vascular hyperpermeability, neuroglial and microvascular pathology, and loss of endogenous repair mechanisms [4,6,7,8]. The development of this complication is a complex, multifactorial process that is influenced by many indirect risk factors [3,6,7,8,9,10,11]. Hence, the development of DR is challenging to predict. Nevertheless, considering the importance and impact of this complication on patients’ quality of life and economic issues, the prediction and prevention of DR is of paramount importance [2,3,4,5,12,13]. Growing evidence from the latest studies suggests that many modifiable risk factors, such as smoking, alcohol consumption, fluctuations in serum glucose level, or hyperlipidemia, play an important role in the DR pathogenesis [2,4,8,14]. This is a crucial observation, which plays an essential role in the prevention of the development of DR. The objective of this cross-sectional analysis was to examine and compare the prevalence of diabetic retinopathy and comorbidities, as well as the clinical characteristics, glycemic control, and prevention patterns in patients with diabetes. Moreover, we tried to evaluate the readiness of patients to use telemedicine technology in chronic care as well as the potential barriers limiting its uptake. We believe that continuous supervision of diabetic patients with telemedicine-based screening can significantly improve treatment outcomes, particularly during the COVID-19 pandemic, when personal contact with the attending physician is remarkably limited. 

## 2. Experimental Section

A cross-sectional study was carried out in July–October 2020 in a sample of 300 participants with diabetes mellitus (types 1 and 2) aged 18 years or older, using a questionnaire-guided interview that included questions about demographic data, disease history, health status in terms of occurrence of symptoms of the disease, utilization of clinical preventive services, willingness to employ telemedical solutions, and health-related behaviors. Participants in this study were outpatients and inpatients from various locations in Poland. The inclusion criteria were as follows: (1) aged 18 years or older, (2) a diabetes diagnosis with no other serious complications, (3) able to read and write in Polish. The research team developed a questionnaire for interviews with diabetic patients using the Diabetes Specific Scale designed by Stanford Patients Education Research Center [15] as a base. The questionnaire was adapted, translated, and pre-tested for use in the Polish population.

The variables of interest in this study are age, gender, urban/rural residence, education, type of diabetes, participants’ body mass index (BMI kg/m^2^), presence of chronic non-communicable diseases such as hypertension, high cholesterol level, obesity, osteoporosis, heart diseases, and renal diseases, use of medication for controlling diabetes and blood pressure, current smoking and alcohol consumption status, vaccination status, foot self-care behavior, use of special diet, physical activity, use of telemedicine solutions to control disease, and factors limiting the use of such solutions.

Data were analyzed using Statistica v.13.3 (TIBCO Software Inc., Palo Alto, CA, USA). Descriptive statistics were calculated for continuous quantitative variables, and the non-parametric significance test (Mann–Whitney U) was used for qualitative variables (nominal and ordinal), the numbers (*n*) and structure indexes (%) were calculated, and chi-square tests of independence were used. Whenever statistical hypothesis testing was used, a *p*-value of less than 0.05 was considered statistically significant. Participants provided their verbal consent at the beginning of the interview. No compensation was provided for participating in this study. This study was approved by the Bioethics Committee of Wroclaw Medical University.

## 3. Results

**Figure 1:**
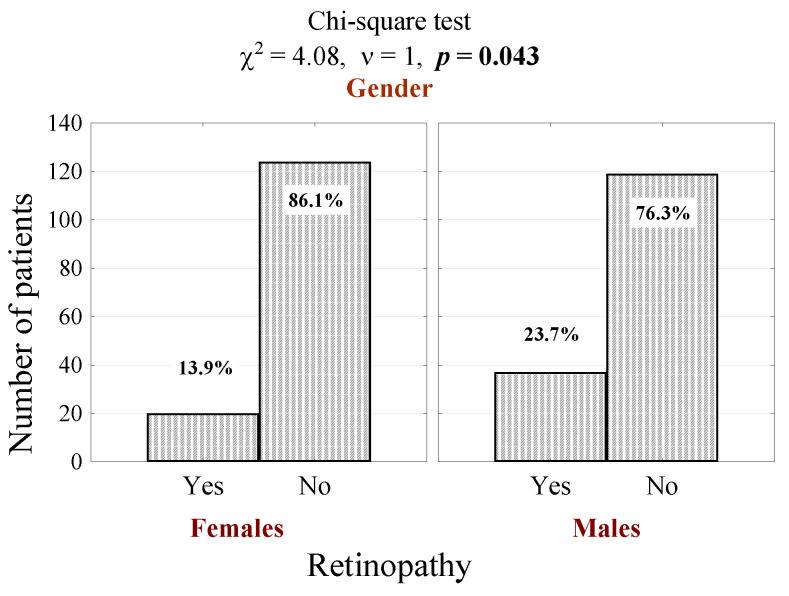
Number (percentage) of patients in groups differing in the presence of retinopathy and gender, and the test of independence.

The cross-sectional analysis included 300 patients (156 male and 144 female) suffering from diabetes type 1 or 2. DR was diagnosed in 57 cases (19%). In the male subgroup, DR was diagnosed in 37 cases (23.7%), and in the female subgroup, in 20 cases (13.9%). Men were found to develop DR significantly more often than women (Figure 1; *p* = 0.043). 

**Figure 2:**
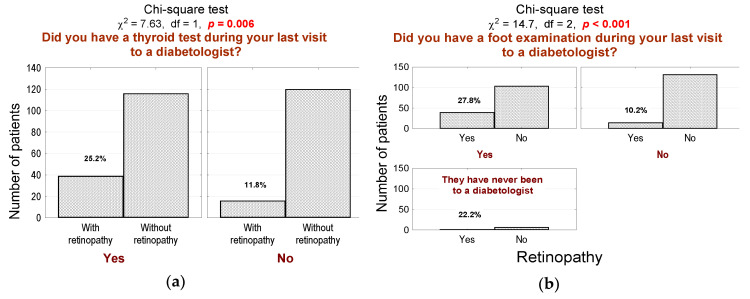
(**a**) Number (*n*) and percentage (%) of patients in groups that differed in the presence of diabetic retinopathy and thyroid examination by a diabetologist at the last visit and the result of the independence test; (**b**) number (*n*) and percentage (%) of patients in groups differing in the presence of diabetic retinopathy and foot examination by a diabetologist at the last visit and the result of the independence test.

Patients with DR are significantly more often under the supervision of medical staff compared to patients without DR. This subgroup more often undergoes physical thyroid examination (Figure 2a; *n* = 39, 68% in the DR group vs. 48% in the non-DR group, *p* = 0.014) and diabetic foot examination (Figure 2b; *n* = 40, 70% in the DR group vs. 43% in the non-DR group, *p* < 0.001) during consultations. 

**Figure 3:**
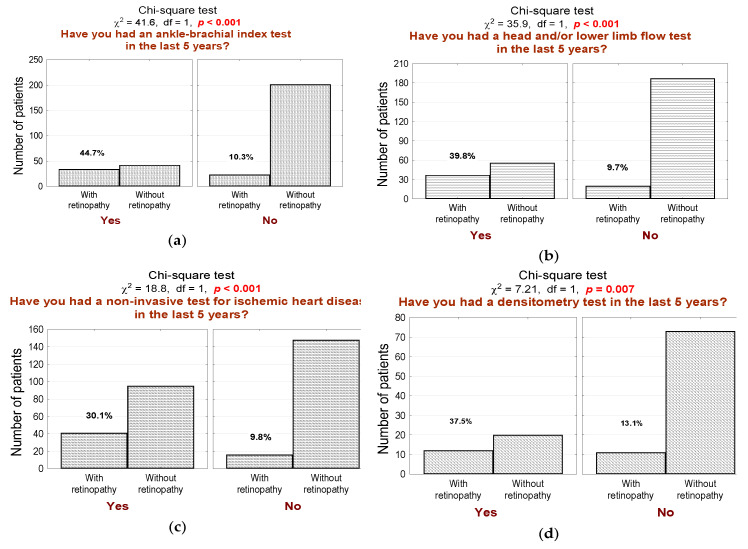
(**a**) Number (*n*) and percentage (%) of patients in groups that differ in the presence of diabetic retinopathy and ankle-brachial index measurement and the test of independence; (**b**) number (*n*) and percentage (%) of patients in groups that differed in the presence of diabetic retinopathy and Doppler ultrasound test of carotid or femoral blood flow measurement and the result of the independence test; (**c**) number (*n*) and percentage (%) of patients in groups differing in the presence of diabetic retinopathy and non-invasive testing for ischemic heart diseases, such as cardiac stress test, stress ECHO test, magnetic resonance imaging of the heart or myocardial perfusion scintigraphy and the test of independence; (**d**) number (*n*) and percentage (%) of patients in groups differing in the presence of diabetic retinopathy and densitometry testing and the result of the independence test.

Moreover, in the last 5 years, the DR subgroup significantly more often had ankle-brachial index measurement (Figure 3a; *n* = 34, 60% in the DR group vs. 17% in the non-DR group, *p* < 0.001), Doppler ultrasound test of carotid or femoral blood flow (Figure 3b; *n* = 37, 65% in the DR group vs. 23% in the non-DR group, *p* < 0.001), non-invasive testing for ischemic heart diseases, such as cardiac stress test, stress ECHO test, magnetic resonance imaging of the heart or myocardial perfusion scintigraphy (Figure 3c; *n* = 41, 72% in the DR group vs. 39% in the non-DR group, *p* < 0.001), and densitometry testing (Figure 3d; *n* = 12, 21% in the DR group vs. 8% in the non-DR group, *p* = 0.007). 

**Figure 4:**
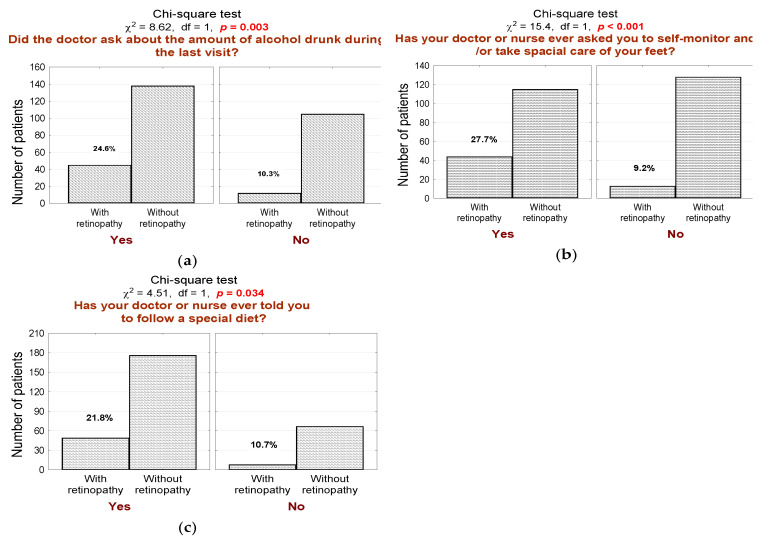
(**a**) Number (*n*) and percentage (%) of patients in groups differing in the presence of diabetic retinopathy and screening for alcohol consumption and the result of the independence test; (**b**) number (*n*) and percentage (%) of patients in groups that differed in the presence of diabetic retinopathy and interviewed about foot self-care behavior and the result of the independence test; (**c**) number (*n*) and percentage (%) of patients in groups differing in the presence of diabetic retinopathy and advice on the use of a special diet and test of independence.

DR subgroup is more often screened for alcohol consumption (Figure 4a; *n* = 45, 79% in the DR group vs. 57% in the non-DR group, *p* = 0.003), interviewed about foot self-care behavior (Figure 4b; *n* = 44, 77% in the DR group vs. 47% in the non-DR group, *p* < 0.001), and advised on the use of a special diet (Figure 4c; *n* = 49, 86% in the DR group vs. 72% in the non-DR group, *p* = 0.034).

**Figure 5:**
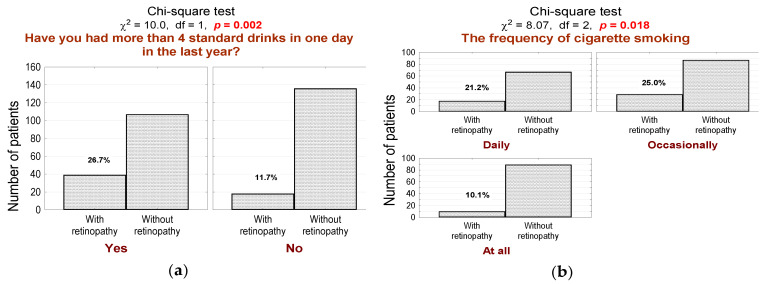
(**a**) Number (*n*) and percentage (%) of patients in groups differing in the presence of diabetic retinopathy and alcohol abuse (more than four standard portions of alcohol in one day in the last period of 12 months), and the result of the independence test; (**b**) number (*n*) and percentage (%) of patients in groups that differ in the presence of diabetic retinopathy and nicotinism, and the test of independence.

Furthermore, patients with DR significantly more often abuse alcohol (more than four standard portions of alcohol in one day in the last period of 12 months, (Figure 5a; *n* = 39, 68% in the DR group vs. 44% in the non-DR group, *p* = 0.002), and more often smoke cigarettes (Figure 5a; *n* = 18, 32% in the DR group vs. 28% in the non-DR group, *p* = 0.018). 

**Figure 6:**
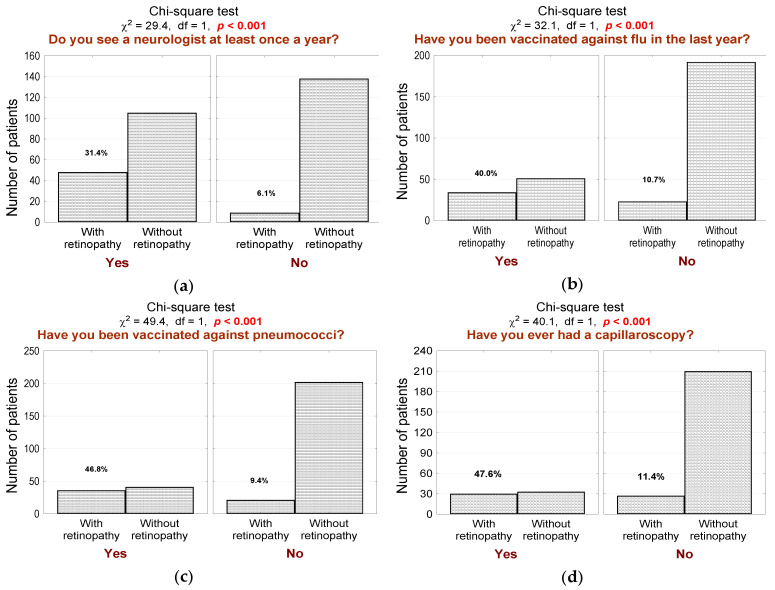
(**a**) Number (*n*) and percentage (%) of patients in groups that differ in the presence of diabetic retinopathy and in the annual neurologist follow-up and the test of independence; (**b**) number (*n*) and percentage (%) of patients in groups differing in the presence of diabetic retinopathy and influenza vaccination in the last year and the test of independence; (**c**) number (*n*) and percentage (%) of patients in groups differing in the presence of diabetic retinopathy and pneumococcal vaccination and the test of independence; (**d**) number (*n*) and percentage (%) of patients in groups differing in the presence of diabetic retinopathy and the capillaroscopy performed, and the result of the independence test.

DR group also had significantly more frequent neurologist consultations (Figure 6a; *n* = 48, 84% in the DR group vs. 43% in the non-DR group, *p* < 0.001), influenza (Figure 6b; *n* = 34, 60% in the DR group vs. 21% in the non-DR group, *p* < 0.001) and pneumococcal (Figure 6c; *n* = 36, 63% in the DR group vs. 17% in the non-DR group, *p* < 0.001) vaccination, resting ECG (*p* = 0.003) and capillaroscopy (Figure 6d; *n* = 30, 53% in the DR group vs. 14% in the non-DR group, *p* < 0.001). 

**Figure 7:**
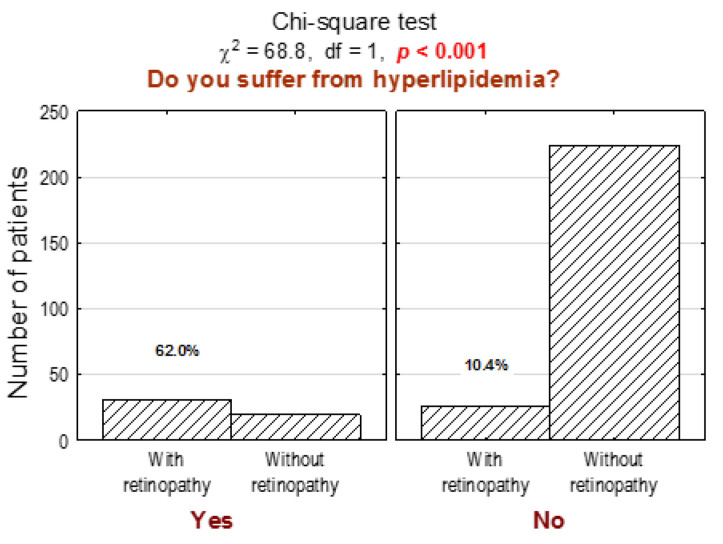
Number (*n*) and percentage (%) of patients in groups differing in the presence of diabetic retinopathy and hyperlipidemia, and the test of independence.

Patients from the DR group more often suffer from hyperlipidemia (Figure 7; *n* = 31, 54% in the DR group vs. 8% in the non-DR group, *p* < 0.001) and are more often on antihypertensive therapy (*p* < 0.001). Moreover, in the DR subgroup, significantly more frequent inadequate diabetic control (too low or too high serum glucose level and consequently hypo- or hyperglycemia symptoms) was observed. Furthermore, DR subgroup patients significantly more often report various signs and symptoms, such as lower extremity pain, dizziness, circulatory system problems, microcirculation disorders, taste and smell impairment, and urinary system infections. Statistical significance and more detailed data about tested participants’ clinical characteristics and preventive care are presented in Tables S1 and S2, respectively. We also asked all participants what sources they utilize to learn about their disease and to mark their answers from 1 to 5, where 1 refers to “definitely not”, and 5 refers to “definitely yes”.

**Figure 8:**
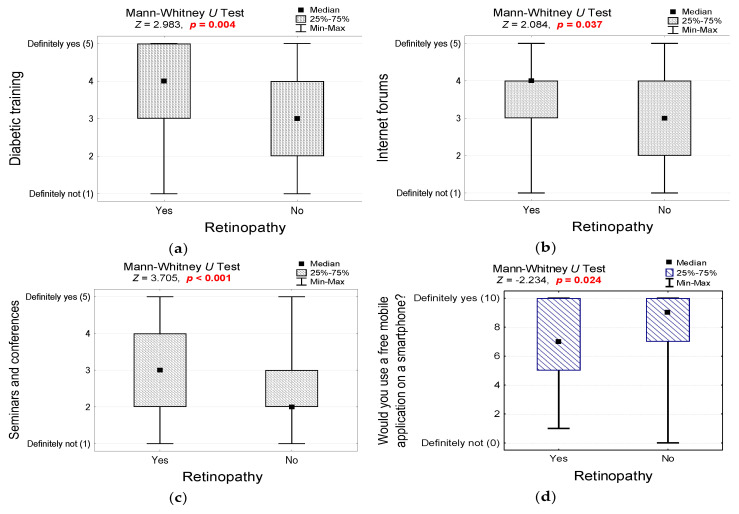
(**a**) Responses to the question about the sources of knowledge about the disease and methods of management (diabetic training) in groups of patients differing in the presence of diabetic retinopathy and the results of the non-parametric test of significance; (**b**) responses to the question about the sources of knowledge about the disease and methods of management (Internet forums, Facebook, etc.) in groups of patients differing in the presence of diabetic retinopathy and the results of the non-parametric test of significance; (**c**) responses to the question about the sources of knowledge about the disease and methods of management (seminars and conferences) in groups of patients differing in the presence of diabetic retinopathy and the results of the non-parametric test of significance; (**d**) responses to the question about the possible use of a free mobile application to monitor the disease in groups of patients differing in the presence of diabetic retinopathy and the result of the non-parametric significance test.

Interestingly, compared to the non-DR group, the DR subgroup patients more often use diabetic training (Figure 8a; 3.6 ± 1.2 vs. to 3.0 ± 1.2, *p* = 0.004), websites and Facebook (Figure 8b; 3.3 ± 1.2 vs. 3.0 ± 1.1, *p* = 0.037), and seminar and conferences (Figure 8c; 3.2 ± 1.3 vs. 2.5 ± 1.2, *p* < 0.001) as their source of information about the disease. We asked questions about the patients’ attitudes toward information technologies to investigate their willingness to utilize telemedical solutions (Table S3). Only 17.3% of all diabetic patients use mobile applications to monitor their disease. However, the patients declare their readiness to use telemedical solutions to track their disease. Interestingly, in the DR subgroup, patients would less often use a mobile application to monitor the disease (Figure 8d; 8.0 ± 2.6 vs. 7.4 ± 2.5 in the non-DR subgroup, *p* = 0.024). The reasons were the inability to use an application (*n* = 6, 29%), lack of trust in the storage of sensitive data (*n* = 12, 57%), and lack of trust in modern technologies (*n* = 3, 14%).

## 4. Discussion

DR is the most frequent and one of the most severe diabetic complications [2,16,17,18,19]. This pathology is considered to be a life-limiting and life-threatening condition both by medical staff and patients [16,17,18,19]. Despite the improvement in the management of diabetes, DR remains the leading cause of visual acuity impairment and blindness worldwide [2,3,14,16]. DR, particularly in its vision-threatening stages, significantly influences both the physical and mental components of quality of life [20]. Since it affects patients’ daily routine and reduces the levels of their independence, the disease is associated with worse life satisfaction scores, depression, and lower income [21]. Patients with DR are more likely to have difficulty maintaining social contact and experience disintegration of their societal lives. Moreover, DR increases the level of anxiety over maintaining friendships or acquaintances or meeting new people because of difficulty identifying faces [22]. Younger individuals with DR report visual impairment as a major limiting factor in finding potential partners and establishing romantic relationships [23].

According to our study, patients from the DR subgroup consider their health state as worse than patients from the non-DR subgroup (*p* = 0.043). This corroborates the previous findings regarding the impact of DR on patients’ quality of life.

It is worth mentioning that visual impairment caused by DR frequently results in unemployment and loss of income [24]. Although it is challenging to segregate the eye cost from the total diabetes healthcare expense, it is estimated that in Germany alone, the financial burden for treating individuals with DR ranged between USD 2.78 and 4.38 billion [25]. Interestingly, a recent study by Sasongko et al. in Indonesia has shown that the projected cost of DR treatment will increase substantially to more than threefold in 2025 [26]. Hence, it is evident that the prevention of DR through effective screening programs is of great importance, not only to improve the quality of life of patients with diabetes but also to reduce the financial burden in the future.

Our results present that DR subgroup patients are significantly more often subjected to various medical tests. This observation is in line with other studies’ findings [1,7,10]. Such conduct is essential because prevention, early diagnosis, and effective treatment of many comorbidities, such as hypertension, hyperlipidemia, or heart disease, exert a significant impact on DR development and progression [9,12,16,27]. We found that 54% of DR patients suffer from hyperlipidemia, and 68% suffer from hypertension. This is significantly more often than in patients in the non-DR group (*p* < 0.001). It emphasizes the importance of preventive screening of such comorbidities and regular monitoring of DR progression. Furthermore, DR is not the only complication of diabetes, as it also leads to various disorders such as chronic kidney disease, lower extremity ischemia, coronary heart disease, neuropathy, stroke, myocardial infarction, depression, and many others [1,3,7,8,9,10,11,28,29]. We found that DR patients significantly more often undergo several medical examinations to diagnose these life-limiting and life-threatening complications. Although we observed statistical significance between both groups in the frequency of such medical tests, unfortunately, not everyone undergoes such necessary testing. For example, only 72% of DR patients undergo non-invasive testing for ischemic heart diseases, 65% undergo a Doppler ultrasound test of carotid or femoral blood flow, and 21% undergo densitometry testing. In the non-DR group, the results were even worse, even though both groups (particularly DR group patients) could benefit from such testing. This suggests that the preventive screening gap is vast, and it is crucial to improve screening coverage, which produces health and economic benefits. We believe that telemedicine solutions bear the potential to fill the gap in preventive screening. Nevertheless, a close relationship between visual and cardiovascular complications justifies a complex disease monitoring approach and treatment.

In our study, we observed that DR subgroup patients were significantly more often screened for alcohol consumption and cigarette smoking during diabetic consultations. This observation underlines the clinical significance of continuous assessment of modifiable risk factors. Patients’ awareness of non-pharmacological factors in the treatment of carbohydrate metabolism disorder is crucial in modulating and ameliorating diabetic complications. Based on our results and the literature, we believe that continuous education may play a significant role in a comprehensive approach to DR management [5,8,30]. Moreover, appropriate education seems to be central in the prevention of the development and progression of DR. Therefore, we believe that DR patients could immensely benefit from it. It is assumed that continuous assessment of modifiable risk factors coupled with constant education is attainable with the use of telemedicine-based surveillance. 

We noticed that in the DR subgroup, patients statistically more often present abnormal glucose serum levels. This observation is of great importance because both hypo- and hyperglycemia are significant risk factors in the development and progression of DR [2,6,7]. Hyperglycemia leads to retinal vascular basement membrane thickening that occurs early in diabetes and is related to hyperglycemia-mediated increases in the production of extracellular matrix proteins fibronectin and collagen, combined with impaired degradation processes [4]. Hypoglycemia, however, contributes to DR development by exacerbating the ischemic retinal injury. Therefore, it is essential to maintain proper glucose serum levels and avoid both hypo- and hyperglycemia. It is reasonable in DR patients to decrease glucose serum levels with antidiabetic drugs with very low capacity to provoke hypoglycemia, like GLP-1RA and SGLT-2 inhibitors. Moreover, hypo- and hyperglycemia are also relevant risk factors for many other diabetes-related complications [2,9]. A telemedical technology reminding about blood glucose measurement could reduce the fluctuations of serum glucose levels and improve treatment outcomes.

Interestingly, we observed that DR patients more often and more willingly take part in diabetes training. Furthermore, they more often draw knowledge from websites and seminars or conferences. This is an observation of great importance because, as mentioned before, education plays a vital role in the management of DR. 

Continuous development of telemedical technologies opens up new opportunities to reach patients suffering from DR. Moreover, it bears the potential to provide better, uninterrupted medical care, which could result in improved treatment outcomes. Telemedicine-based DR screening programs are increasingly popular and proved to be successful in increasing screening rates for DR [31]. The approach bears the potential to move the screening locus from the specialty eye care setting to the primary care setting [32]. This is particularly important, as about half of patients fail to keep ophthalmology appointments within 1 year during the follow-up [33]. It is well-established that such a gap in preventive care can lead to clinically significant vision loss. Previous studies, as well as our findings, highlight the need for a more robust tracking and recall system for patients failing to keep their ophthalmology appointment. In our study, we identified that the patients with DR are less prone to use the mobile application to monitor the disease than non-DR patients (*p* = 0.024). The reported reasons were the inability to use an application (*n* = 6, 29%), lack of trust in the storage of sensitive data (*n* = 12, 57%), and lack of trust in modern technologies (*n* = 3, 14%). However, the patients indicate that the uptake of telemedical technology could be increased if it was recommended by their personal physician and provided without additional costs. Our findings are particularly important, as accumulating evidence suggests that telemedicine-based screening programs may have a significant impact on reducing the healthcare burden from the growing diabetes epidemic [34,35]. Mansberger et al. have shown in a randomized controlled trial that patients using telemedicine solutions were more likely to receive a diabetic retinopathy screening examination when compared with the traditional surveillance group [36]. The evidence suggests that telemedicine in the form of teleophthalmology bears the potential to improve accessibility to DR screening programs by increasing compliance with preventive screening as well as reducing the incidence of vision-threatening complications on a large scale. Further studies of telemedicine technology to monitor DR, in particular using automated retinal image evaluation and mobile phone-based teleophthalmology platforms, holds significant future promise. Utilization of telemedicine in diabetes eye care will allow us to extend access to professional care and integrate DR surveillance into the patient’s total healthcare.

There are several limitations to our study. First, the study design restricted our ability to assess the evolutionary process of DR. Second, the cross-sectional character of this study impeded any conclusion about causal relations, making it challenging to draw firm assumptions about the direction of exposure-outcome associations. Third, the limited sample size yielded broad confidence intervals with the risk of overlooking associated characteristics. Fourth, the possibility of a recall bias cannot be ruled out in self-reports of self-management, making the findings of this investigation reliant upon the accuracy of the patient’s self-evaluation. 

In conclusion, DR is a severe, mutilating disease that causes enormous medical and economic burdens. A multifaceted medical approach to these patients is amply justified and indispensable, considering that many comorbidities usually coexist with DR and influence disease development and progression. Hypo- and hyperglycemia play a crucial role in developing DR; therefore, it is essential to maintain the correct glucose serum level. Considering that hypoglycemia is also an important risk factor in DR development, we believe antidiabetic drugs with limited capacity to provoke hypoglycemia (GLP-1RA and SGLT-2 inhibitors) should be used to treat DR patients. Proper education is essential in preventing and slowing down the development of DR. Evolution of telemedicine offers a possibility of inexpensive, continuous monitoring of the disease that could improve treatment outcomes and thus lighten the health and economic burden of DR and other diabetic complications. Our observations emphasize DR’s perception as a complex disease in which education and continuous surveillance, particularly with telemedicine-based screening programs, are crucial to yield progress in chronic care. Telemedicine is of substantial significance during the COVID-19 pandemic when traditional medical consultations are increasingly limited. Our observations and the accumulating evidence suggest that utilizing telemedicine-based care to monitor and manage diabetes and diabetic complications is essential in improving health outcomes. 

## Data Availability

The data presented in this study are available on request from the corresponding author. The data are not publicly available due to ethical restrictions.

## Supplementary Materials

The following are available online at https://www.mdpi.com/2077-0383/10/2/249/s1, Table S1: Clinical characteristics of patients in the diabetic retinopathy (DR) group and non-DR group. Number (*n*) and percentage (%) of answers; Table S2: Preventive care of patients in the DR group and non-DR group. Number (*n*) and percentage (%) of answers; Table S3: Attitude to telemedical solutions and sources utilized to learn about the disease in the DR group and non-DR group. Number (*n*) and percentage (%) of answers.
