# Supplementary material for: Clinical Characteristics, Preventive Care and Attitude to Telemedicine among Patients with Diabetic Retinopathy: A Cross-Sectional Study
Source: J Clin Med. 2021 Jan 12;10(2):249. doi: 10.3390/jcm10020249 (PMC7827263; doi:10.3390/jcm10020249)
Supplement: Supplementary file 1 [file jcm-10-00249-s001.pdf]

Table S1. Clinical characteristics of patients in the diabetic retinopathy (DR) group and non-DR group. Number (*n*) and percentage (%) of answers.

Tested Parameter	All Participants N = 300		DR Group N = 57		Non-DR Group N = 243		Test Result *p*
	n	%	*n*	%	*n*	%	
The occurrence of hypoglycemia symptoms, such as sweating, fatigue, tremor, anxiety, hunger or headache during last month							0.006
0	52	17.3	6	11.5	46	88.5	
1–3 times	148	49.3	21	14.2	127	85.8	
4–6 times	83	27.7	23	27.7	60	72.3	
7–12 times	13	4.3	6	46.2	7	53.8	
More than 12 times	4	1.3	1	25.0	3	75.0	
The occurrence of severe incidents caused by hypoglycemia such as loss of consciousness or immediate help need during the period of last 12 months							<0.001
0	137	45.7	13	9.5	124	90.5	
1–3 times	108	36.0	25	23.1	83	76.9	
4–6 times	42	14.0	16	38.1	26	61.9	
7–12 times	9	3.0	3	33.3	6	66.7	
More than 12 times	4	1.3	0	0.0	4	100.0	
The occurrence of hyperglycemia symptoms, such as thirst, dryness of skin or mouth, decreased appetite, nausea, or fatigue during last month							<0.001
0	69	23.0	8	11.6	61	88.4	
1–3 days	150	50.0	24	16.0	126	84.0	
4–6 days	55	18.3	19	34.5	36	65.5	
7–12 days	16	5.3	1	6.3	15	93.8	
More than 12 days	10	3.3	5	50.0	5	50.0	
Frequency of occurrence of too high serum glucose level caused by illness or infection during the period of last 12 months							0.001
1—Never	64	21.3	5	7.8	59	92.2	
2—Rarely	66	22.0	9	13.6	57	86.4	
3—Sometimes	143	47.7	31	21.7	112	78.3	
4—Quite often	17	5.7	8	47.1	9	52.9	
5—Often	10	3.3	4	40.0	6	60.0	
M ± SD	2.5 ± 1.0		2.9 ± 1.0		2.4 ± 1.0		<0.001
Me [Q1; Q3]	3 [2; 3]		3 [3; 3]		3 [2; 3]		
Min–Max	1–5		1–5		1–5		
Frequency of occurrence of too high serum glucose level caused by nervousness or anger during the period of last 12 months							0.476
1—Never	34	11.3	3	8.8	31	91.2	
2—Rarely	58	19.3	10	17.2	48	82.8	
3—Sometimes	123	41.0	25	20.3	98	79.7	
4—Quite often	40	13.3	10	25.0	30	75.0	
5—Often	45	15.0	9	20.0	36	80.0	
M ± SD	3.0 ± 1.2		3.2 ± 1.1		3.0 ± 1.2		0.148
Me [Q1; Q3]	3 [2; 4]		3 [3; 4]		3 [2; 4]		
Min–Max	1–5		1–5		1–5		
Frequency of occurrence of too high serum glucose level caused by the administration of the wrong dose of medicine during the period of last 12 months							<0.001
1—Never	167	55.7	17	10.2	150	89.8	
2—Rarely	37	12.3	8	21.6	29	78.4	
3—Sometimes	62	20.7	18	29.0	44	71.0	
4—Quite often	22	7.3	8	36.4	14	63.6	
5—Often	12	4.0	6	50.0	6	50.0	
M ± SD	1.9 ± 1.2		2.6 ± 1.3		1.8 ± 1.1		<0.001
Me [Q1; Q3]	1 [1; 3]		3 [1; 3]		1 [1; 3]		
Min–Max	1–5		1–5		1–5		

Frequency of occurrence of too high serum glucose level caused by the administration of the wrong type of food during the period of last 12 months							0.002
1—Never	34	11.3	3	8.8	31	91.2	
2—Rarely	56	18.7	10	17.9	46	82.1	
3—Sometimes	148	49.3	26	17.6	122	82.4	
4—Quite often	35	11.7	15	42.9	20	57.1	
5—Often	27	9.0	3	11.1	24	88.9	
M ± SD	2.9 ± 1.1		3.1 ± 0.9		2.8 ± 1.1		0.050
Me [Q1; Q3]	3 [2; 3]		3 [3; 4]		3 [2; 3]		
Min–Max	1–5		1–5		1–5		
Frequency of occurrence of too high serum glucose level caused by the administration of too much food during the period of last 12 months							0.134
1—Never	38	12.7	3	7.9	35	92.1	
2—Rarely	45	15.0	7	15.6	38	84.4	
3—Sometimes	144	48.0	27	18.8	117	81.3	
4—Quite often	48	16.0	14	29.2	34	70.8	
5—Often	25	8.3	6	24.0	19	76.0	
M ± SD	2.9 ± 1.1		3.2 ± 1.0		2.9 ± 1.1		0.014
Me [Q1; Q3]	3 [2; 3]		3 [3; 4]		3 [2; 3]		
Min–Max	1–5		1–5		1–5		
Frequency of occurrence of too high serum glucose level caused by less physical activity during the period of last 12 months							<0.001
1—Never	46	15.3	4	8.7	42	91.3	
2—Rarely	50	16.7	4	8.0	46	92.0	
3—Sometimes	145	48.3	24	16.6	121	83.4	
4—Quite often	33	11.0	12	36.4	21	63.6	
5—Often	26	8.7	13	50.0	13	50.0	
M ± SD	2.8 ± 1.1		3.5 ± 1.1		2.7 ± 1.0		<0.001
Me [Q1; Q3]	3 [2; 3]		3 [3; 4]		3 [2; 3]		
Min–Max	1–5		1–5		1–5		
Frequency of occurrence of too high serum glucose level caused by stress during the period of last 12 months							0.060
1—Never	31	10.3	2	6.5	29	93.5	
2—Rarely	42	14.0	11	26.2	31	73.8	
3—Sometimes	128	42.7	21	16.4	107	83.6	
4—Quite often	44	14.7	7	15.9	37	84.1	
5—Often	55	18.3	16	29.1	39	70.9	
M ± SD	3.2 ± 1.2		3.4 ± 1.2		3.1 ± 1.2		0.130
Me [Q1; Q3]	3 [3; 4]		3 [3; 5]		3 [3; 4]		
Min–Max	1–5		1–5		1–5		
Frequency of occurrence of too low serum glucose level caused by illness or infection during the period of last 12 months							<0.001
1—Never	102	34.0	10	9.8	92	90.2	
2—Rarely	72	24.0	11	15.3	61	84.7	
3—Sometimes	89	29.7	21	23.6	68	76.4	
4—Quite often	28	9.3	10	35.7	18	64.3	
5—Often	9	3.0	5	55.6	4	44.4	
M ± SD	2.2 ± 1.1		2.8 ± 1.2		2.1 ± 1.0		<0.001
Me [Q1; Q3]	2 [1; 3]		3 [2; 4]		2 [1; 3]		
Min–Max	1–5		1–5		1–5		
Frequency of occurrence of too low serum glucose level caused by nervousness or anger during the period of last 12 months							0.013
1—Never	74	24.7	8	10.8	66	89.2	
2—Rarely	63	21.0	11	17.5	52	82.5	
3—Sometimes	104	34.7	18	17.3	86	82.7	
4—Quite often	35	11.7	13	37.1	22	62.9	
5—Often	24	8.0	7	29.2	17	70.8	
M ± SD	2.6 ± 1.2		3.0 ± 1.2		2.5 ± 1.2		0.003
Me [Q1; Q3]	3 [2; 3]		3 [2; 4]		3 [1; 3]		

Min–Max	1–5		1–5		1–5		
Frequency of occurrence of too low serum glucose level caused by the administration of the wrong dose of medicine during the period of last 12 months							0.010
1—Never	135	45.0	14	10.4	121	89.6	
2—Rarely	48	16.0	11	22.9	37	77.1	
3—Sometimes	77	25.7	22	28.6	55	71.4	
4—Quite often	20	6.7	4	20.0	16	80.0	
5—Often	20	6.7	6	30.0	14	70.0	
M ± SD	2.1 ± 1.2		2.6 ± 1.2		2.0 ± 1.2		0.001
Me [Q1; Q3]	2 [1; 3]		3 [2; 3]		2 [1; 3]		
Min–Max	1–5		1–5		1–5		
Frequency of occurrence of too low serum glucose level caused by the administration of the wrong type of food during the period of last 12 months							0.015
1—Never	75	25.0	7	9.3	68	90.7	
2—Rarely	57	19.0	13	22.8	44	77.2	
3—Sometimes	123	41.0	28	22.8	95	77.2	
4—Quite often	26	8.7	2	7.7	24	92.3	
5—Often	19	6.3	7	36.8	12	63.2	
M ± SD	2.5 ± 1.1		2.8 ± 1.1		2.5 ± 1.1		0.063
Me [Q1; Q3]	3 [2; 3]		3 [2; 3]		3 [1; 3]		
Min–Max	1–5		1–5		1–5		
Frequency of occurrence of too low serum glucose level caused by the administration of too much food during the period of last 12 months							0.140
1—Never	64	21.3	7	10.9	57	89.1	
2—Rarely	61	20.3	9	14.8	52	85.2	
3—Sometimes	118	39.3	26	22.0	92	78.0	
4—Quite often	33	11.0	10	30.3	23	69.7	
5—Often	24	8.0	5	20.8	19	79.2	
M ± SD	2.6 ± 1.2		2.9 ± 1.1		2.6 ± 1.2		0.017
Me [Q1; Q3]	3 [2; 3]		3 [2; 4]		3 [2; 3]		
Min–Max	1–5		1–5		1–5		
Frequency of occurrence of too low serum glucose level caused by less physical activity during the period of last 12 months							0.225
1—Never	85	28.3	10	11.8	75	88.2	
2—Rarely	49	16.3	10	20.4	39	79.6	
3—Sometimes	111	37.0	22	19.8	89	80.2	
4—Quite often	38	12.7	11	28.9	27	71.1	
5—Often	17	5.7	4	23.5	13	76.5	
M ± SD	2.5 ± 1.2		2.8 ± 1.2		2.4 ± 1.2		0.033
Me [Q1; Q3]	3 [1; 3]		3 [2; 4]		3 [1; 3]		
Min–Max	1–5		1–5		1–5		
Frequency of occurrence of too low serum glucose level caused by skipping the meal during the period of last 12 months							0.674
1—Never	62	20.7	8	12.9	54	87.1	
2—Rarely	54	18.0	11	20.4	43	79.6	
3—Sometimes	125	41.7	25	20.0	100	80.0	
4—Quite often	37	12.3	9	24.3	28	75.7	
5—Often	22	7.3	4	18.2	18	81.8	
M ± SD	2.7 ± 1.2		2.8 ± 1.1		2.6 ± 1.2		0.262
Me [Q1; Q3]	3 [2; 3]		3 [2; 3]		3 [2; 3]		
Min–Max	1–5		1–5		1–5		
Frequency of occurrence of too low serum glucose level caused by stress during the period of last 12 months							0.036
1—Never	66	22.0	5	7.6	61	92.4	
2—Rarely	49	16.3	9	18.4	40	81.6	
3—Sometimes	111	37.0	27	24.3	84	75.7	
4—Quite often	44	14.7	7	15.9	37	84.1	
5—Often	30	10.0	9	30.0	21	70.0	

M ± SD	2.7 ± 1.2		3.1 ± 1.1		2.7 ± 1.2		0.019
Me [Q1; Q3]	3 [2; 3]		3 [3; 4]		3 [1; 3]		
Min–Max	1–5		1–5		1–5		
The pain of lower extremities during physical exercises							<0.001
Yes	141	47.0	41	29.1	100	70.9	
No	159	53.0	16	10.1	143	89.9	
Circulatory system problems, such as retrosternal pain, dyspnoea, palpitations							0.003
Yes	129	43.0	35	27.1	94	72.9	
No	171	57.0	22	12.9	149	87.1	
Dizziness							0.009
Yes	172	57.3	42	24.4	130	75.6	
No	128	42.7	15	11.7	113	88.3	
Microvascular disturbances							<0.001
Yes	95	31.7	38	40.0	57	60.0	
No	205	68.3	19	9.3	186	90.7	
Taste and smell impairment							<0.001
Yes	72	24.0	33	45.8	39	54.2	
No	228	76.0	24	10.5	204	89.5	
Problems with correct recognition of stimuli acting on the skin							<0.001
Yes	65	21.7	33	50.8	32	49.2	
No	235	78.3	24	10.2	211	89.8	
Recurrent urinary tract infections							<0.001
Yes	102	34.0	32	31.4	70	68.6	
No	198	66.0	25	12.6	173	87.4	
Use of diuretics, ACEI or ARB							<0.001
Yes	93	31.0	39	41.9	54	58.1	
No	207	69.0	18	8.7	189	91.3	
Presence of hyperlipidemia							<0.001
Yes	50	16.7	31	62.0	19	38.0	
No	250	83.3	26	10.4	224	89.6	

M—mean, Me—median, SD—standard deviation, Q1—quartile 1, Q3—quartile 3.

Table S2. Preventive care of patients in the diabetic retinopathy (DR) group and non-DR group. Number (*n*) and percentage (%) of answers.

Survey Questions	All Participants *N* = 300		DR Group *N* = 57		Non-DR Group *N* = 243		Test Result *p*
Have you undergone a physical thyroid examination during your last diabetic consultation?							0.014
Yes	155	51.7	39	25.2	116	74.8	
No	136	45.3	16	11.8	120	88.2	
Never been on diabetic consultation	9	3.0	2	22.2	7	77.8	
Have you undergone a foot examination during your last diabetic consultation?							<0.001
Yes	144	48.0	40	27.8	104	72.2	
No	147	49.0	15	10.2	132	89.8	
Never been on diabetic consultation	9	3.0	2	22.2	7	77.8	
Have your medical doctor asked you about alcohol consumption during your last consultation?							0.003
Yes	183	61.0	45	24.6	138	75.4	
No	117	39.0	12	10.3	105	89.7	
Has it happened to you to drink more than 4 standard portions of alcohol in one day (1 standard portion of alcohol is 12.5ml of pure ethyl alcohol) in the last 12 months?							0.002
Yes	146	48.7	39	26.7	107	73.3	
No	154	51.3	18	11.7	136	88.3	
How often do you smoke cigarettes?							0.018
Everyday	85	28.3	18	21.2	67	78.8	
From time to time	116	38.7	29	25.0	87	75.0	
Never	99	33.0	10	10.1	89	89.9	

Do you have a control neurological consultation at least once a year?							<0.001
Yes	153	51.0	48	31.4	105	68.6	
No	147	49.0	9	6.1	138	93.9	
Have you undergone densitometry testing in the period of the last 5 years?							0.007
Yes	32	27.6	12	37.5	20	62.5	
No	84	72.4	11	13.1	73	86.9	
Have you got an influenza vaccination in the last year?							<0.001
Yes	85	28.3	34	40.0	51	60.0	
No	215	71.7	23	10.7	192	89.3	
Have you got a pneumococcal vaccination in the last year?							<0.001
Yes	77	25.7	36	46.8	41	53.2	
No	223	74.3	21	9.4	202	90.6	
Have you undergone ankle-brachial index measurement in the period of the last 5 years?							<0.001
Yes	76	25.3	34	44.7	42	55.3	
No	224	74.7	23	10.3	201	89.7	
How often do you undergo resting electrocardiogram (ECG)?							0.003
Every half-year	46	15.3	17	37.0	29	63.0	
Every year	113	37.7	22	19.5	91	80.5	
Only when presenting symptoms	107	35.7	15	14.0	92	86.0	
Never	34	11.3	3	8.8	31	91.2	
Have you undergone non-invasive testing for ischemic heart diseases, such as cardiac stress test, stress echocardiogram test (ECHO), magnetic resonance imaging of the heart, or myocardial perfusion scintigraphy during the last 5 years?							<0.001
Yes	136	45.3	41	30.1	95	69.9	
No	164	54.7	16	9.8	148	90.2	
Have you undergone a Doppler ultrasound test of carotid or femoral blood flow during the last 5 years?							<0.001
Yes	93	31.0	37	39.8	56	60.2	
No	207	69.0	20	9.7	187	90.3	
Have you ever undergone a capillaroscopy?							<0.001
Yes	63	21.0	30	47.6	33	52.4	
No	237	79.0	27	11.4	210	88.6	
Do you examine your feet during a bath every day?							0.014
Yes	164	54.7	40	24.4	124	75.6	
No	136	45.3	17	12.5	119	87.5	
Do your medical doctor or nurse ever recommend foot self-care behavior?							<0.001
Yes	159	53.0	44	27.7	115	72.3	
No	141	47.0	13	9.2	128	90.8	
Has your doctor or nurse ever told you to follow a special diet?							0.034
Yes	225	75.0	49	21.8	176	78.2	
No	75	25.0	8	10.7	67	89.3	

Table S3. Attitude *to* telemedical solutions and sources utilized to learn about the disease in the diabetic retinopathy (DR) group and non-DR group. Number (*n*) and percentage (%) of answers.

Survey Questions	All Participants *N* = 300		DR Group *N* = 57		Non-DR Group *N* = 243		Test Result
	**n**	**%**	***n***	**%**	***n***	**%**	***p***
Do you currently use any mobile application to monitor your state of health?							0.308
Yes	52	17.3	13	25.0	39	75.0	
No	248	82.7	44	17.7	204	82.3	
What would have a positive effect on deciding to use a free mobile application to monitor your state of health? (0 – definitely not, and 10– definitely yes)							
1. The application would be free of charge and easily available							
M ± SD	8.0 ± 2.4		7.3 ± 2.4		8.1 ± 2.4		0.006
Me [Q1; Q3]	9 [7; 10]		7 [5; 10]		9 [7; 10]		
Min–Max	0–10		2–10		0–10		
2. The application would be recommended by the National Health Fund or Health Insurance Company							
M ± SD	7.5 ± 2.6		7.2 ± 2.7		7.6 ± 2.6		0.293

Me [Q1; Q3]	8 [6; 10]		8 [5; 10]		8 [6; 10]		
Min–Max	0 - 10		1 - 10		0 - 10		
3. The application would be recommended as a part of private medical care packages							
M ± SD	7.0 ± 2.7		6.8 ± 2.6		7.0 ± 2.7		0.418
Me [Q1; Q3]	7 [5; 9]		7 [5; 9]		8 [5; 9]		
Min–Max	0–10		0–10		0–10		
4. The application would be recommended by your personal physician							
M ± SD	7.5 ± 2.4		7.4 ± 2.3		7.5 ± 2.4		0.631
Me [Q1; Q3]	8 [6; 10]		8 [5; 10]		8 [6; 10]		
Min–Max	0–10		0–10		0–10		
5. The application would guarantee a discount in the insurance company on the purchase of a life or health insurance policy							
M ± SD	7.2 ± 2.8		7.0 ± 2.5		7.3 ± 2.9		0.291
Me [Q1; Q3]	8 [5; 10]		7 [5; 10]		8 [5; 10]		
Min–Max	0–10		0–10		0–10		
If there was a free of charge mobile application which could, in an intelligent way, conduct monitoring of your state of health and, based on medical knowledge, recommend you proper proceeding, would you use it?							0.024
M ± SD	7.9 ± 2.6		7.4 ± 2.5		8.0 ± 2.6		
Me [Q1; Q3]	9 [7; 10]		7 [5; 10]		9 [7; 10]		
Min–Max	0–10		0–10		0–10		
Why would you not use such an application?	*n*	%	*n*	%	*n*	%	
1. Lack of trust in modern technologies	15	5.0%	3	20.0	12	80.0	1.000
2. lack of trust in the storage of sensitive data	40	13.3	12	30.0	28	70.0	0.091
3. Inability to use application	15	5.0	6	40.0	9	60.0	0.033
4. Lack of a smartphone or computer	2	0.7	0	0.0	2	100.0	1.000
5. Other cause	1	0.3	0	0.0	1	100.0	1.000
6. Habit	4	1.3	0	0.0	4	100.0	1.000
7. have familiar physician	11	3.7	2	18.2	9	81.8	1.000
8. have familiar laboratory	1	0.3	0	0.0	1	100.0	1.000
9. have a medical package that enables free service provision	8	2.7	2	25.0	6	75.0	0.650
10. Free service provision by the National Health Fund	17	5.7	4	23.5	13	76.5	0.540
Sources utilized to learn about the disease (1 refers to—definitely not, and 5 refers to—definitely yes)							
1. Diabetic training							0.004
M ± SD	3.1 ± 1.2		3.6 ± 1.2		3.0 ± 1.2		
Me [Q1; Q3]	3 [2; 4]		4 [3; 5]		3 [2; 4]		
Min–Max	0–5		0–5		0–5		
2. Websites and Facebook							0.037
M ± SD	3.1 ± 1.2		3.3 ± 1.2		3.0 ± 1.1		
Me [Q1; Q3]	3 [2; 4]		4 [3; 4]		3 [2; 4]		
Min–Max	0–5		0–5		0–5		
3. Seminar and conferences							<0.001
M ± SD	2.6 ± 1.2		3.2 ± 1.3		2.5 ± 1.2		
Me [Q1; Q3]	3 [2; 3]		3 [2; 4]		2 [2; 3]		
Min–Max	0–5		0–5		0–5		

M—mean, Me—median, SD—standard deviation, Q1—quartile 1, Q3—quartile 3.
